# Organization of hyaluronic acid molecules in solutions

**DOI:** 10.1557/s43580-024-00875-4

**Published:** 2024-05-20

**Authors:** Ferenc Horkay, Peter J. Basser

**Affiliations:** Section on Quantitative Imaging and Tissue Sciences, Eunice Kennedy Shriver National Institute of Child Health and Human Development, National Institutes of Health, Bethesda, MD 20892 USA

## Abstract

The organization of hyaluronic acid (HA) solutions was investigated by small angle neutron scattering (SANS). It was demonstrated that HA formed clusters greater than several hundred nanometers. The SANS response revealed a weak correlation peak, corresponding to ordering over a distance scale of about 80 nm. At higher wave vectors, the SANS response indicated the presence of rod-like structures. Addition of calcium ions only weakly affects the structure of the HA solution, but significantly reduces its osmotic pressure. The osmotic component of the SANS response is in good agreement with the intensity obtained from direct osmotic pressure measurements. The results are consistent with the rigid structure of the HA molecule.

**Graphical abstract:**

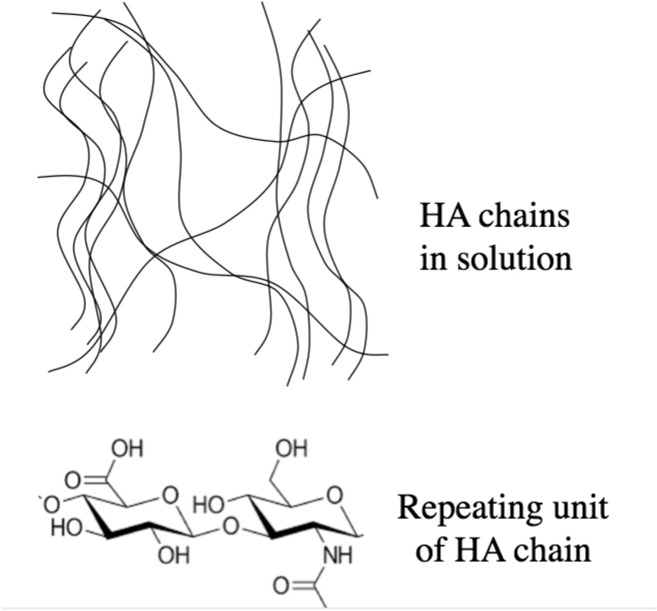

## Introduction

Hyaluronic acid (HA) is a major constituent of the extracellular matrix (ECM). Among the many biological functions of the ECM in various tissues, it plays role in tissue development and regulates the response of the tissue during injury, repair, and regeneration. HA forms large complexes with the bottlebrush shape aggrecan molecules [1, 2]. In cartilage large aggrecan/HA, complexes provide osmotic resistance under external load. HA is also an essential component of the synovial fluid, which has important role in biolubrication [3]. HA is a relatively stiff biopolymer, mainly due to the electrostatic repulsion between the charged groups on the polymer backbone. Its persistence length is close to 100 Å [4].

Many biophysical studies have been made on HA solutions [5–8]. Scaling relations have been derived for the osmotic pressure in the semi-dilute concentration regime in the presence of monovalent and divalent counter-ions. It was found that the osmotic pressure was governed by the ionic strength of the salt solution, and the HA solution exhibited remarkably stability in the presence of salts even well beyond the physiological concentration range. The latter behavior is different from that of typical synthetic and biological polyelectrolytes (e.g., polyacrylic acid and DNA). In general, polyelectrolyte solutions phase separate in the presence of multivalent counter-ions, because the repulsive electrostatic interactions among the polyelectrolyte chains are screened by the ions [9–12].

However, previous investigations have not addressed the relationship between the thermodynamic properties and the organization of HA molecules in solutions at physiologically relevant ion concentrations. In the physiological environment of HA, both mono- and divalent ions are present. It is not clear what changes occur in the presence of multivalent counter-ions in HA solutions, in which the polyelectrolyte chains consist of relatively rigid elements.

We studied the dynamic properties of HA solutions by dynamic light scattering (DLS) and rheology [7, 13]. The DLS correlation function revealed two distinct relaxation rates, separated by about one order of magnitude. The fast mode is a diffusive process that describes the decay of the thermodynamic concentration fluctuations. The slow mode indicates the presence of slowly moving domains (large clusters, aggregates) in the solutions. Our rheological measurements have indicated that HA solutions exhibit characteristics of entangled polymer solutions and shear thinning. It was also found that addition of NaCl and CaCl_2_ salts reduced the low-shear viscosity of HA solutions but neither salt-induced phase separation nor calcium ion bridge formation was observed [13].

In the present work, we investigate the effect of ions on the thermodynamic (osmotic) concentration fluctuations in HA solutions. Previous scattering and osmotic measurements made by our group on numerous polymer systems conclusively showed that the scattering intensity governed by the thermodynamic concentration fluctuations is separable from that due to larger-scale clusters [14–17]. We intend to determine how the presence of calcium ions affects the amplitude of the osmotic concentration fluctuations in HA solutions. In this study we explore the structure of nearly physiological HA solutions at length scales intermediate between the macroscopic and the short-range atomic structure of the molecule, i.e., between 1 μm and 1 nm. To determine structures in this spatial range, small angle neutron scattering (SANS) is used.

## Experimental section

### Sample preparation

Solutions of sodium hyaluronate (HA, Sigma *M*_w_ = 1.2 10^6^) were prepared in D_2_O containing 100-mM NaCl and various amounts of CaCl_2_. The concentration of the HA was varied in the range 0.5–6 wt%. The sodium chloride concentration (100 mM) was identical in all samples.

### Small angle neutron scattering measurements

SANS measurements were made on the NG3 instrument at NIST, Gaithersburg MD. Sample cells with quartz windows and 2-mm path length were used. SANS measurements were made at three sample-detector distances, 1.35 m, 4 m, and 13.1 m with an incident wavelength of 8 Å at 25° ± 0.1 °C. This configuration corresponds to the transfer wave vector range 2.8 10^−3^ Å^−1^ < *q* < 0.35 Å^−1^. After azimuthal averaging, corrections were made for incoherent background, detector response, and cell window scattering.

### Osmotic pressure measurements

The osmotic pressure of the HA solutions was measured by a method described previously [7]. The HA solutions were equilibrated with poly(vinyl alcohol) gel filaments of known osmotic swelling pressure. At equilibrium, the swelling pressure of the gel is equal to the osmotic pressure of the surrounding solution.

All measurements were made at 25 ± 0.1 °C.

## Theory

### Small angle neutron scattering

In the semi-dilute concentration range, in the absence of large-scale associations, both the scattering and the osmotic properties of a solution of flexible polymer chains can be described by a single correlation length ξ. In such solutions, the scattering intensity arising from the osmotic concentration fluctuations is given as follows [18]:

1 $$I_{{os}} \left( q \right) = \Delta \rho ^{2} \frac{{k_{B} Tc}}{{\partial \Pi /\partial c}}\frac{1}{{\left( {1 + q^{2} \xi ^{2} } \right)}},$$

where *q* = 4*π*/λ sin(*θ*/2) is the transfer wave vector, λ is the wavelength of the incident radiation, and θ is the angle of observation. Δ*ρ*^2^ is the neutron contrast factor between the polymer and the solvent, *c* is the polymer concentration, *k*_*B*_ is the Boltzmann constant, and *T* is the absolute temperature. In the presence of large-scale structures, e.g., polymer aggregates and clusters, these objects contribute to the total scattering intensity

2 $$I\left( q \right){\text{ }} = I_{{os}} \left( q \right){\text{ }} + I_{{ag}} \left( q \right),$$

where the second term depends on the size of the aggregates. If these objects are very large, scattering from their surfaces or internal structure dominates. In many cases, their contribution to the scattering response can be expressed as a power law of the form.

3 $$I_{{ag}} \left( q \right){\text{ }} = Aq^{{ - m}},$$

where *A* is a constant and the exponent *m* is defined by 3 ≤ *m* < 4 for surface scattering and *m* < 3 for volume fractal behavior. Equations 1, 2, and 3 make it possible to separate the osmotically driven concentration fluctuations (solution-like component) from the total scattering intensity [14].

## Results and discussion

### Static scattering properties

**Fig. 1:**
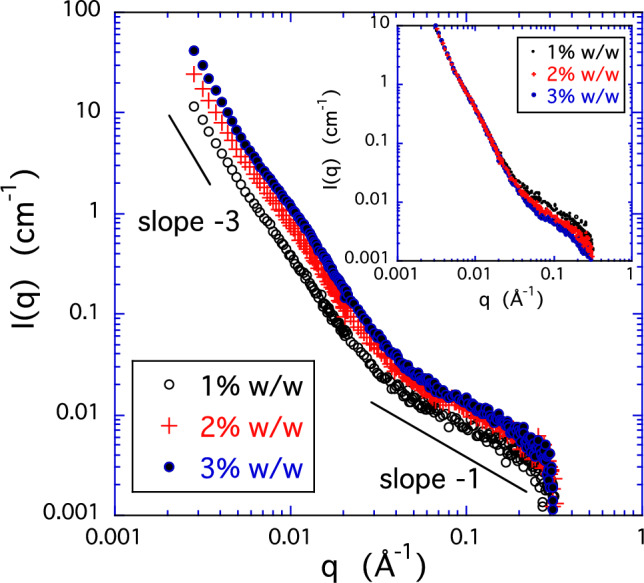
SANS intensity *I*(*q*) from HA solutions at three concentrations, Inset: SANS profile of the same HA solutions, normalized by the polymer concentration

Figure 1 shows the SANS intensity *I*(*q*) from HA solutions at three concentrations, *c* = 1, 2, and 3% w/w. In the scattering profiles three regions can be distinguished. For *q* < 0.006 Å^−1^ power law behavior with a slope slightly greater than − 3 is observed, corresponding to surface scattering. According to the language of surface fractals, these surfaces are so rough (*D*_s_ = 6 − *m* = 3 − *ε*, where *D*_s_ is the surface fractal dimension and in this case 0.1 < *ε* < 0.3) that they are at the limit of being volume fractals. The power law shows no sign of weakening even at the lowest *q* value. We attribute the cause of this scattering to loose domains of size exceeding several hundred nanometers. These are apparently equilibrium structures observed in previous studies on polyelectrolyte solutions [12, 14].

**Fig. 2:**
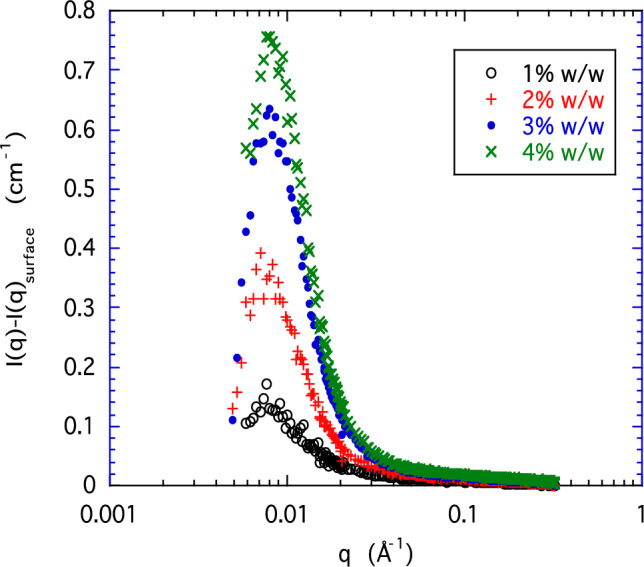
Excess scattering in the low-*q* region after subtraction of power law background

At *q* ≈ 0.008 Å^−1^, a deviation from the power law behavior in the form of a weak shoulder can be identified. This feature is more clearly resolved if the contribution from surface scattering is subtracted from the total intensity (Fig. 2). The peak indicates the presence of objects separated on average by a distance *d* ≈ 2*π*/*q*_max_, where *q*_max_ is the position of the peak. From Fig. 2 it follows that the value of *d* is approximately 80 nm. The intensity of the peak increases with the HA concentration, but its position does not vary appreciably in the concentration range explored in the present experiment. This peak resembles the well-known “polyelectrolyte peak” observed in many polyelectrolyte solutions at low salt concentration, although the scale is much larger in the present instance where the salt concentration is relatively high. Analysis of the Guinier region immediately beyond the peak yields an apparent radius of gyration of these elements of roughly 13 nm. In the upper *q*-range of Fig. 1, the slope of the *I*(*q*) plots is approximately − 1, which is the signature of rod-like elements and is consistent with the relatively stiff nature of the HA chain. The high-*q* end of the curves is terminated by a downturn, corresponding to a scale on the order of the chain cross-sectional dimension. Specifically, a Guinier plot of this part of the spectrum yields for the cross-sectional radius approximately 0.25 nm, corresponding to a single HA strand.

In the low-*q* region of the inset in Fig. 1, the data points fall on the same curve, i.e., the intensity is proportional to the polymer concentration. This finding indicates that the large clusters are distinct and approximately non-interacting and also that their structure is independent of the concentration [12, 19]. On the other hand, the concentration dependence of the intensity in the *q*-region above 0.03 Å^−1^, is different. In this region, the scattering intensity is primarily governed by the osmotic concentration fluctuations and is thus sensitive to the polymer and salt concentrations as well as the geometry of the fluctuating elements. *I*(*q*) exhibits a weaker than linear dependence on concentration, varying approximately as *c*^0.5^. This behavior is different from the thermodynamic response of semi-dilute solutions of flexible polymers. In polymer solutions in general, the scattering intensity in this region is governed by the thermal concentration fluctuations associated with the macroscopic osmotic pressure. The corresponding scattering intensity, *I*_*os*_(*q*), depends on the concentration and the structure according to

4 $$I_{{os}} \left( q \right) \propto c^{{(3 - 2D_{f} )/(3 - D_{f} )}},$$

where the fractal dimension *D*_f_ = 5/3 applies in excluded volume conditions [18]. In that situation, *I*_*os*_(*q*) is expected to decrease with concentration as *c*^−0.25^. For rigid rods, however, *D*_*f*_ = 1 and hence *I*_*os*_(*q*) should vary as *c*^0.5^, which is consistent with the present observation. This exponent is also consistent with the theoretical expectation for stiff polymer molecules [20]. We conclude that the scattering from HA solution in this region is due to thermodynamic concentration fluctuations involving rod-like chain segments.

**Fig. 3:**
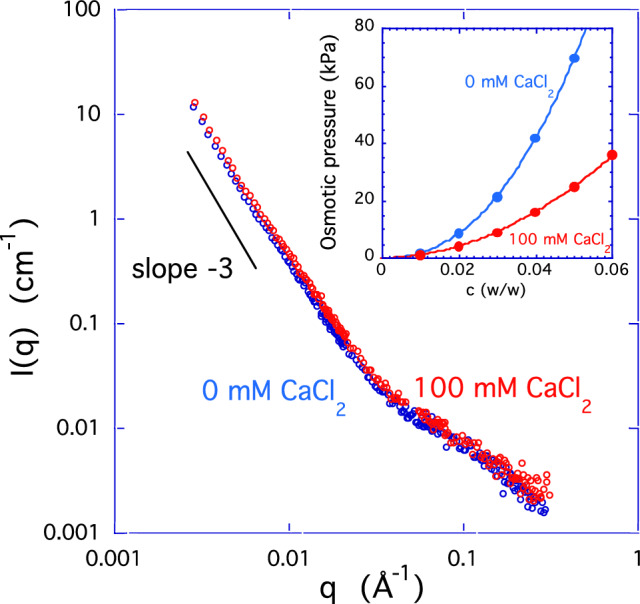
SANS spectra from sample *c* = 1% w/w in 100-mM NaCl, without CaCl_2_, and with 100-mM CaCl_2_. Inset: Concentration dependence of the osmotic pressure of HA solutions

**Fig. 4:**
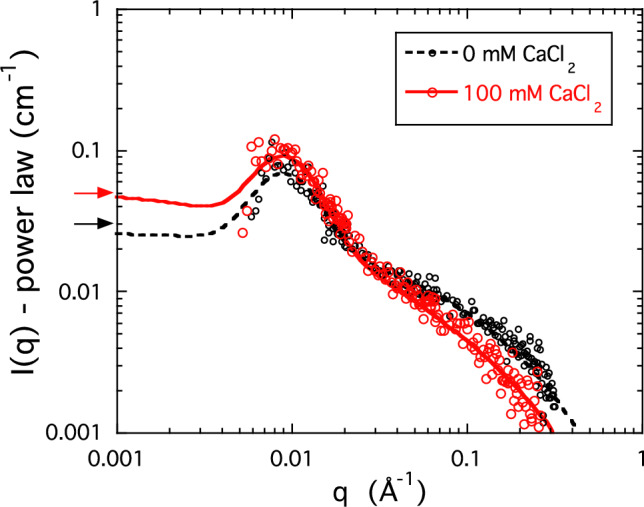
Excess scattering in the low-*q* region for 1% w/w HA solutions after subtraction of power law background; O: 100-mM NaCl and 0-mM CaCl_2_; O: 100-mM NaCl and 100-mM CaCl_2_. Arrows show the scattering intensities at *q* = 0 Å^−1^ calculated from osmotic pressure measurements

As noted in the Introduction, the natural physiological environment of HA contains calcium ions, which, in solutions of high-molecular weight linear polyelectrolytes, might be expected to have a strong effect on the shape and size of the molecule. Figure 4 shows the SANS response from two 1% w/w solutions of HA in 100-mM NaCl, one without CaCl_2_ and the other containing 100-mM CaCl_2_. At low and intermediate values of *q* the calcium-containing and the calcium-free samples display closely similar behavior, while at high *q* a small excess intensity can be discerned in the latter case. The inset in Fig. 3 shows the concentration dependence of the osmotic pressure of the same HA solutions. 100-mM CaCl_2_ reduces the osmotic pressure by a factor of roughly 2. We note that the presence of large objects (clusters, aggregates) only weakly affects the osmotic pressure of polymer solutions.

### Effect of calcium ions of the thermodynamic concentration fluctuations

To estimate the effect of ions on the thermodynamic response requires the separation of the scattering intensity due to osmotic concentration fluctuations from the intensity scattered by larger-scale structures.

The effect of calcium ions on the high-*q* region of the SANS response is shown in Fig. 4, where, as in Fig. 2, the initial low-*q* power law has been subtracted from the total scattering intensity. The resulting residual signals from two 1% w/w solutions of HA, one in 100-mM NaCl and the other containing in addition 100-mM CaCl_2_, are compared in a logarithmic representation. The absence of any significant difference between the two correlation peaks demonstrates that calcium ions do not modify the separation distance between the HA clusters. It may be expected that the thermodynamic fluctuations become dominant in the higher *q* range of the SANS response, where the scattering from the clusters vanishes. As noted earlier, the *q*^-1^ dependence of the scattering intensity in this region is the signature of rod-like structures. If these structures correspond to the osmotic fluctuations in the present system, then, by analogy with Eq. 1, the scattering intensity of the semi-dilute polymer solution can be described empirically by [21]

5 $$I_{{os}} \left( q \right) = \Delta \rho ^{2} \frac{{k_{B} Tc}}{{\partial \Pi /\partial c}}\frac{1}{{\left( {1 + qL} \right)\left( {1 + q^{2} r_{c}^{2} } \right)}}.$$

*L* being the length and *r*_c_ is the cross-section of the element, where *qr*_*c*_ ≤ 1. The total SANS response is thus the sum of the thermodynamic contribution described by Eq. 5 and the surface scattering in addition to the correlation peak visible in Fig. 2.

To model the system semiquantitatively, we assume that the structure that gives rise to the correlation peak is independent of the osmotic fluctuations and can be approximated by a lognormal function centered on the peak. The SANS signal, from which the surface scattering has been subtracted, then becomes

6 $$I\left( q \right) - I_{{surface}} \left( q \right) = \Delta \rho ^{2} \frac{{k_{B} Tc}}{{\partial \Pi /\partial c}}\frac{1}{{\left( {1 + qL} \right)\left( {1 + q^{2} r_{c}^{2} } \right)}} + a\exp \left[ { - \frac{{\left( {\ln q - \ln q_{{\max }} } \right)}}{b}^{2} } \right],$$

where *a* and *b* are fitting parameters. In the low-*q* limit, expression 6 tends to Δρ^2^*k*_*B*_*Tc*/(∂Π/∂*c*). The continuous lines in Fig. 4 are the fits of Eq. 6 to the two datasets, where *r*_*c*_ is taken to be 0.25 nm and *q*_max_ = 9 × 10^−3^ Å^−1^. Although the fits of Eq. 6 are subject to a large error owing to the noise associated with the difference spectra and the limited *q*-range of the decisive data, the signals from the two samples are clearly distinct. Also shown in the same figure are the intensities estimated from osmotic pressure measurements, expressed in terms of the SANS intensity by means of Eq. 1. The horizontal arrows at the left axis of Fig. 4 show the intensities from the osmotic pressure data. It can be seen that increasing the concentration of calcium ions increases the amplitude of the thermodynamic concentration fluctuations. This model of the osmotic contribution to the scattering intensity is consistent with our interpretation above that the thermodynamically fluctuating elements in the HA solutions are rod like.

## Conclusion

SANS measurements made on HA solutions containing 100-mM NaCl reveal a hierarchical organization composed of large clusters of size greater than several hundred nanometers. These clusters generate surface fractal scattering of dimensionality *D*_s_ ≈ 2.7–2.9. A correlation peak is observed in the SANS spectra whose position corresponds to a scale of about 80 nm, which does not vary with either the concentration of the HA solution or with the ionic composition. Specifically, addition of calcium ions in stoichiometric excess leaves the structure of the HA solution practically unmodified, but simply reduces the solvent quality. The SANS response contains an osmotic component which is in good agreement with the scattering intensity estimated from osmotic pressure measurements.

The role of charged biopolymers in biological processes makes it important to understand their behavior, particularly in physiological or near-physiological salt conditions. For example, structure formation in these systems is mediated by the charge density of the polymer, as well as by the concentration and valence of the ions. The ability of charged polymers to respond to changes in solvent conditions (e.g., ionic concentration, ion valence) is strongly influenced by chain rigidity, chain hydrophobicity, and chemical details (including specific interactions) but there is little understanding of how these factors affect solution thermodynamic properties (e.g., osmotic pressure). Clearly, further work is needed to better understand the unique structural and osmotic behavior of important biological polymers.

## Data Availability

The data that support the findings of this study are available from the corresponding author upon reasonable request.
